# Supplementary material for: Developing a QSPR Model of Organic Carbon Normalized Sorption Coefficients of Perfluorinated and Polyfluoroalkyl Substances
Source: Molecules. 2022 Aug 31;27(17):5610. doi: 10.3390/molecules27175610 (PMC9457706; doi:10.3390/molecules27175610)
Supplement: Supplementary file 1 [file molecules-27-05610-s001.zip › molecules-1858245-supplementary.pdf]

Developing a QSPR Model of Organic Carbon Normalized Sorption Coefficients of Perfluorinated and Polyfluoroalkyl Substances

Lan Jiang [1], Yue Xu [1,*], Xiaoyu Zhang [2,*], Bingfeng Xu [1], Ximeng Xu [1] and Yixing Ma [3]

[1] Faculty of Civil Engineering and Mechanics, Kunming University of Science and Technology, Kunming 650500, China
[2] Yunnan Research Academy of Eco-environmental Sciences, Kunming 650034, China
[3] Faculty of Environmental Science and Engineering, Kunming University of Science and Technology, Kunming 650500, China
* Correspondence: xuyue@kust.edu.cn (Y.X.); todzhang1999@hotmail.com (X.Z.)

List of contents:

1. The five guidelines for QSPR construction.
2. Descriptor selection.
3. Model validation.
4. The application domain.
Table S1. The values of log K_{oc} for PFASs.
Table S2. Description of the descriptors generated from Multiwfn.
Table S3. Statistical parameters of QSPR model.
Table S4. The observed and predicted log K_{oc} values of PFASs. The values of the descriptors used in the QSPR models.

1. The five guidelines for QSPR construction.

The OECD principles are the best possible outline of the essential points to be addressed while developing reliable and reproducible QSPR/QSAR models. The principles were formulated by QSAR experts in a meeting held in Setúbal, Portugal, in March 2002 as the guidelines for the validation of QSPR/QSAR models, in particular for regulatory purposes. These principles were later approved by the OECD member countries, QSAR and regulatory communities at the 37th Joint Meeting of the Chemicals Committee and Working Party on Chemicals, Pesticides and Biotechnology in November 2004.

The five guidelines adopted by the OECD denoting validity of QSPR/QSAR model are as follows:

- Principle 1—A defined endpoint.
- Principle 2—An unambiguous algorithm.
- Principle 3—A defined domain of applicability.
- Principle 4—Appropriate measures of goodness of fit, robustness and predictivity.
- Principle 5—A mechanistic interpretation, if possible.

2. Descriptor selection

The descriptors of the 62 PFASs computed by Multiwfn are shown in Table S2. This study uses the following methods to select descriptors to develop a QSPR model.

(1) The constant descriptors, almost–constant descriptors and descriptors with missing values were deleted. These descriptors include: q_{C+}, q_{F-}, q_{O-}, q_H, q_S, q_N, R_r, D_{max}, D_{min}, AD_C, AD_F, D_S, R_S, MPP, SDP.

(2) Correlation analysis was performed on the remaining 47 descriptors. For descriptors with a correlation coefficient higher than 0.9, only one molecular descriptor with a high correlation coefficient with log K_{oc} was retained. After processing, 29 descriptors are obtained, including MW, $V_{s,max}$, $V_{s,min}$, ρ, S^+, S^-, $V_{mean,all}$, $V_{mean,positive}$, $V_{mean,negative}$, $V^2_{positive}$, $V^2_{negative}$, S_{polar}, MPI, $ALIE_{max}$, $ALIE_{min}$, E_{LUMO}, $E_{HOMO\text{-}LUMO\ gap}$, ODI_{HOMO}, ODI_{LUMO}, VIP, X_M, E_H, ω_{cubic}, μ, μ^2, μ^3, r^2, V^{vdw}, β.

(3) Based on SPSS 26 software, using the above 29 descriptors as independent variables and log K_{oc} as the dependent variable to perform stepwise linear regression, the following QSPR models with different numbers of descriptors are obtained:

$$\log K_{oc} = 6.377 \times 10^{-3}\, MW + 0.107 \tag{1}$$

$$\log K_{oc} = 6.469 \times 10^{-3}\, MW - 1.800\, \mu + 1.527 \tag{2}$$

$$\log K_{oc} = 6.764 \times 10^{-3}\, MW - 2.082\, \mu - 0.388\, E_{LUMO} + 0.769 \tag{3}$$

$$\log K_{oc} = 7.334 \times 10^{-3}\, MW - 1.705\, \mu - 0.956\, E_{LUMO} - 1.398\, ALIE_{max} + 24.100 \tag{4}$$

The statistical parameters of different QSPR models are shown in Table S3. After comparison, we selected the QSPR model with the largest adjusted coefficient of determination (R^2_{adj}) and the smallest root mean square error ($RMSE$) as the final model developed for this study. The model contained four molecular descriptors: molecular weight (MW), dipole moment (μ), the lowest unoccupied molecular orbital energy (E_{LUMO}), and the maximum average local ionization energy on the molecular surface ($ALIE_{max}$).

3. Model validation

3.1 Internal validation

Internal validation of a QSPR model is performed based on the molecules used in the model development. We used the internal validation index (the multiple correlation coefficient of leave–one–out cross–validation, Q^2_{LOO}) to internally validate the model.

According to the divided training set and validation set, we applied leave–one–out cross–validation for 18 modeling predictions. For each modeling prediction, one sample is selected as the unknown sample, the remaining 17 samples are used as the training set to establish a calibration model, and the obtained model is used to predict the selected unknown sample. This is repeated 18 times, so that each sample is used as the test set for a prediction in turn. Finally, Q^2_{LOO} is obtained according to the following calculation formula.

3.2 External validation

The external validation ensures the predictability and applicability of the developed QSPR model for the

prediction of untested molecules.

According to the divided training set and validation set, we apply the QSPR model established by the training set to predict the 4 samples in the validation set. The external validation indicators (Q^2_{F1}, Q^2_{F2}, Q^2_{F3}) is obtained according to the following calculation formula.

$$Q^2_{\mathrm{LOO}} = 1 - \frac{\sum(Y_{\mathrm{obs(train)}} - Y_{\mathrm{pred(train)}})^2}{\sum(Y_{\mathrm{obs(train)}} - \bar{Y}_{\mathrm{train}})^2}$$

$$Q^2_{\mathrm{F1}} = 1 - \frac{\sum(Y_{\mathrm{obs(test)}} - Y_{\mathrm{pred(test)}})^2}{\sum(Y_{\mathrm{obs(test)}} - \bar{Y}_{\mathrm{train}})^2}$$

$$Q^2_{\mathrm{F2}} = 1 - \frac{\sum(Y_{\mathrm{obs(test)}} - Y_{\mathrm{pred(test)}})^2}{\sum(Y_{\mathrm{obs(test)}} - \bar{Y}_{\mathrm{test}})^2}$$

$$Q^2_{\mathrm{F3}} = 1 - \frac{\left[\sum(Y_{\mathrm{obs(test)}} - Y_{\mathrm{pred(test)}})^2\right]/n_{\mathrm{ext}}}{\left[\sum(Y_{\mathrm{obs(train)}} - \bar{Y}_{\mathrm{train}})^2\right]/n_{\mathrm{train}}}$$

In the above equation, $Y_{\mathrm{obs(train)}}$ is the observed value of the compound in the training set, while $Y_{\mathrm{obs(test)}}$ is the observed value of the compound in the test set; $Y_{\mathrm{pred(train)}}$ is the predicted value of the compound in the training set, while $Y_{\mathrm{pred(test)}}$ is the predicted value of the compound in the test set; $\bar{Y}_{\mathrm{train}}$ is the mean of all compound observations in the training set, while $\bar{Y}_{\mathrm{test}}$ is the mean of all compound observations in the test set; n_{train} is the number of all compounds in the training set, while n_{test} is the number of all compounds in the test set.

4. The application domain

The applicability domain (AD) of the developed models was characterized by the Williams plot. In the Williams plots of standardized residuals (δ) versus leverage values (h), chemicals with the absolute values of standardized residual $|\delta| > 3$ were identified as outliers. The standardized residual (δ) was calculated by:

$$\delta = \frac{y_i - \hat{y}_i}{\sqrt{\sum_{i=1}^{n}(y_i - \hat{y}_i)^2/(n-A-1)}}$$

where y_i and $\hat{y}_i$ are the observed value and predicted value for the i-th compound, respectively; n is the number of compounds and A is the number of descriptors.

The measure of how far a chemical is from the domain of applicability of a model is its leverage value (Hat), h, defined as:

$$h = x_i^T (X^T X)^{-1} x_i$$

where x_i is the descriptor vector of the i-th compound; $\mathrm{x}_\mathrm{i}^\mathrm{T}$ is the transpose of x_i; X is the descriptor matrix and X^T is the transpose of X. The limit of application domain was quantified as the warning leverage value (h^*), expressed as:

$$h^* = 3(k+1)/n$$

where k is the number of predictor variables used in the model. If a compound in the training set has $h > h^*$, it means that the compound is very influential on the model. If a compound in the test set has $h > h^*$, then the compound is structurally distant from the compounds used in the training set.

Table S1. The values of log K_{oc} for PFASs.

ID	Nomenclature	Abbreviations	log K_{oc} [b]									Avg.
1	perfluorobutanoic acid	PFBA	1.88[1]									1.88
2	perfluoroheptanoic acid	PFPeA	1.37[1]	1.71[2]								1.54
3	perfluorohexanoic acid	PFHxA	1.31[1]	2.08[2]								1.70
4	perfluoroheptanoic acid [a]	PFHpA	1.63[1]	2.45[2]								2.04
5	perfluorooctanoic acid	PFOA	1.89[1]	2.82[2]								2.36
6	perfluorononanoic acid	PFNA	2.36[1]	3.19[2]								2.78
7	perfluorodecanoic acid	PFDA	2.96[1]	3.56[2]								3.26
8	perfluoroundecanoic acid	PFUnDA	3.56[1]	3.93[2]								3.75
9	perfluorododecanoic acid	PFDoDA	4.30[2]									4.30
10	perfluorotridecanoic acid [a]	PFTrDA	4.67[2]									4.67
11	perfluorotetradecanoic acid	PFTeDA	5.04[2]									5.04
12	perfluorobutane sulfonic acid	PFBS	1.79[1]	1.93[2]	1.62[3]	1.42[4]	2.00[5]					1.75
13	perfluorohexane sulfonic acid [a]	PFHxS	2.05[1]	2.67[2]	2.29[3]	2.29[4]	2.40[5]	2.20[6]				2.32
14	perfluoroheptane sulfonic acid	PFHpS	2.90[6]									2.90
15	perfluorooctane sulfonic acid	PFOS	2.80[1]	3.41[2]	3.26[3]	3.62[4]	2.80[5]	3.70[6]	4.40[7]	3.55[7]	2.68[8]	3.28
			2.57[8]									
16	perfluorodecane sulfonic acid	PFDS	3.66[8]	3.53[8]								3.60
17	perfluorooctane sulfonamide	PFOSA	4.28[3]	3.84[4]								4.06
18	6:6 perfluoroalkyl phosphinic acid	6:6 PFPiA	4.93[4]									4.93
19	8:2 perfluoroakyl unsaturated carboxylate	8:2 FTUCA	4.00[7]	4.20[7]								4.10
20	n-ethyl perfluorooctane sulfonamidoacetic acid	N-EtFOSAA	3.49[8]	3.23[8]								3.36
21	6:2 chlorinated polyfluorinated ether sulfonate [a]	6:2 Cl-PFAES	2.60[5]									2.60
22	6:2 fluorotelomer phosphate diester	6:2 diPAP	4.51[4]									4.51

Notes: [a] Chemicals were in the test set, and others were in the training set；[b] Superscripts indicate the source publication.

Table S2. Description of the descriptors generated from Multiwfn.

ID	Descriptor	Description	Unit	The basis set level of the calculation
1	MW	Molecular weight	Da	B3LYP/def2-TZVP
2	TE	Total energy	Hartree	B3LYP/def2-TZVP
3	$V_{s,max}$	The maximum value of molecular surface potential	eV	B3LYP/def2-TZVP
4	$V_{s,min}$	The minimum value of molecular surface potential	eV	B3LYP/def2-TZVP
5	V	Molecular volume	$Bohr^3$	B3LYP/def2-TZVP
6	ρ	Density	g/cm^3	B3LYP/def2-TZVP
7	S	Molecular surface area	$Bohr^2$	B3LYP/def2-TZVP
8	S^+	The surface area of the region where the molecular electrostatic potential is positive	$Bohr^2$	B3LYP/def2-TZVP
9	S^-	The surface area of the region where the molecular electrostatic potential is negative	$Bohr^2$	B3LYP/def2-TZVP
10	$V_{mean,all}$	Overall average value of molecular electrostatic potential	kcal/mol	B3LYP/def2-TZVP
11	$V_{mean,positive}$	The average value of the positive region of the molecular electrostatic potential	kcal/mol	B3LYP/def2-TZVP
12	$V_{mean,negative}$	The average value of the negative region of the molecular electrostatic potential	kcal/mol	B3LYP/def2-TZVP
13	V^2_{all}	Variance of the overall area of the molecular electrostatic potential	$(kcal/mol)^2$	B3LYP/def2-TZVP
14	$V^2_{positive}$	Variance in the positive region of the molecular electrostatic potential	$(kcal/mol)^2$	B3LYP/def2-TZVP
15	$V^2_{negative}$	Variance in the negative region of the molecular electrostatic potential	$(kcal/mol)^2$	B3LYP/def2-TZVP
16	π	Average deviation of electrostatic potential on molecular surfaces	a.u	B3LYP/def2-TZVP
17	MPI	Molecular polarity index	eV	B3LYP/def2-TZVP
18	$S_{non-polar}$	Non-polar surface area	$Angstrom^2$	B3LYP/def2-TZVP
19	S_{polar}	Polar surface area	$Angstrom^2$	B3LYP/def2-TZVP
20	$ALIE_{max}$	The maximum average local ionization energy on the molecular	eV	B3LYP/def2-TZVP
21	$ALIE_{min}$	The minimum average local ionization energy on the molecular	eV	B3LYP/def2-TZVP
22	q_{C+}	The most positive net atomic charge on a carbon atom	a.u	B3LYP/def2-TZVP

ID	Descriptor	Description	Unit	The basis set level of the calculation
23	q_{F-}	The most negative net atomic charge on a fluorine atom	a.u	B3LYP/def2-TZVP
24	q_{O-}	The most negative net atomic charge on an oxygen atom	a.u	B3LYP/def2-TZVP
25	q_H	The most positive net atomic charge on a hydrogen atom	a.u	B3LYP/def2-TZVP
26	q_S	Net atomic charge on sulfur atom	a.u	B3LYP/def2-TZVP
27	q_N	Net atomic charge on nitrogen atoms	a.u	B3LYP/def2-TZVP
28	E_{HOMO}	The highest occupied molecular orbital energy	eV	B3LYP/def2-TZVP
29	E_{LUMO}	The lowest unoccupied molecular orbital energy	eV	B3LYP/def2-TZVP
30	$E_{HOMO\text{-}LUMO\ gap}$	The energy difference between the highest occupied molecular orbital and the lowest unoccupied molecular orbital	eV	B3LYP/def2-TZVP
31	ODI_{HOMO}	The delocalization index of the highest occupied molecular orbital	/	B3LYP/def2-TZVP
32	ODI_{LUMO}	The delocalization index of the lowest unoccupied molecular orbital	/	B3LYP/def2-TZVP
33	VIP	Vertical ionization potential	eV	B3LYP/def2-TZVP
34	VEA	Vertical electron affinity	eV	B3LYP/def2-TZVP
35	X_M	Mulliken electronegativity	eV	B3LYP/def2-TZVP
36	U	Chemical potential	eV	B3LYP/def2-TZVP
37	E_H	Hardness	eV	B3LYP/def2-TZVP
38	E_S	Softness	eV^{-1}	B3LYP/def2-TZVP
39	ω	Electrophilic index	eV	B3LYP/def2-TZVP
40	VIP_{second}	Second vertical ionization energy	eV	B3LYP/def2-TZVP
41	ω_{cubic}	Stricter electrophilic index	eV	B3LYP/def2-TZVP
42	N	Nucleophilicity index	eV	B3LYP/def2-TZVP
43	TN	The sum of the nucleophilic superdelocalization of all atoms	$Hartree^{-1}$	B3LYP/def2-TZVP
44	TE	The sum of the electrophilic superdelocalization of all atoms	$Hartree^{-1}$	B3LYP/def2-TZVP
45	μ	Dipole moment	a.u	B3LYP/def2-TZVPD
46	μ^2	Quadrupole moment	a.u	B3LYP/def2-TZVPD
47	μ^3	Octapole moment	a.u	B3LYP/def2-TZVPD

ID	Descriptor	Description	Unit	The basis set level of the calculation
48	r^2	Electronic space range	a.u	B3LYP/def2-TZVPD
49	V^{vdw}	The minimum point of the molecular van der Waals potential	kcal/mol	B3LYP/def2-TZVPD
50	I	Isotropic average polarizability	a.u	B3LYP/def2-TZVPD
51	V_I	Isotropic average polarizability volume	$Angstrom^3$	B3LYP/def2-TZVPD
52	P_a	Polarzability anisotropy	a.u	B3LYP/def2-TZVPD
53	β	Magnitude of first hyperpolarizability	a.u	B3LYP/def2-TZVPD
54	R_r	Rotation radius	Angstrom	B3LYP/def2-TZVP
55	D_{max}	Maximum distance between atoms	Angstrom	B3LYP/def2-TZVP
56	D_{min}	Minimum distance between atoms	Angstrom	B3LYP/def2-TZVP
57	AD_C	Atomic distance closest to geometric center	Angstrom	B3LYP/def2-TZVP
58	AD_F	Atomic distance farthest from geometric center	Angstrom	B3LYP/def2-TZVP
59	D_S	System diameter	Angstrom	B3LYP/def2-TZVP
60	R_S	System radius	Angstrom	B3LYP/def2-TZVP
61	*MPP*	Molecular flatness parameter	/	B3LYP/def2-TZVP
62	*SDP*	Span out of plane	/	B3LYP/def2-TZVP

Table S3. Statistical parameters of QSPR model.

Model	R^2	R^2_{adj}	*RMSE*	Q^2_{LOO}	Q^2_{F1}	Q^2_{F2}	Q^2_{F3}
(2)	0.831	0.821	0.471	0.783	0.755	0.723	0.746
(2)	0.885	0.869	0.402	0.826	0.937	0.929	0.935
(3)	0.922	0.905	0.342	0.847	0.922	0.912	0.919
(4)	0.962	0.950	0.212	0.920	0.961	0.955	0.959

Notes: R^2: coefficient of determination; R^2_{adj}: adjusted coefficient of determination; *RMSE*: root mean square error; Q^2_{LOO}: multiple correlation coefficient of leave-one-out cross-validation; Q^2_{F1}, Q^2_{F2}, and Q^2_{F3}: external validation indicators.

Table S4. The observed and predicted log K_{oc} values of PFASs. The values of the descriptors used in the QSPR models.

ID	Nomenclature	Abbreviations	Descriptors				log K_{oc}	
			MW	*μ*	E_{HOMO}	$ALIE_{max}$	Observed	Predicted
1	perfluorobutanoic acid	PFBA	214.04	0.7732	−1.8057	17.6830	1.88	1.36
2	perfluoroheptanoic acid	PFPeA	264.05	0.7817	−1.8066	17.7145	1.54	1.67
3	perfluorohexanoic acid	PFHxA	314.05	0.7880	−1.8134	17.7384	1.70	2.00
4	perfluoroheptanoic acid	PFHpA	364.06	0.7918	−1.8156	17.7575	2.04	2.33
5	perfluorooctanoic acid	PFOA	414.07	0.7960	−1.8162	17.7837	2.36	2.65
6	perfluorononanoic acid	PFNA	464.08	0.7976	−1.8121	17.7929	2.78	3.00
7	perfluorodecanoic acid	PFDA	514.08	0.7988	−1.8195	17.8160	3.26	3.34
8	perfluoroundecanoic acid	PFUnDA	564.09	0.8034	−1.8128	17.8179	3.75	3.69
9	perfluorododecanoic acid	PFDoDA	614.10	0.7999	−2.0626	17.8160	4.30	4.31
10	perfluorotridecanoic acid	PFTrDA	664.11	0.8074	−1.8156	17.8063	4.67	4.44
11	perfluorotetradecanoic acid	PFTeDA	714.11	0.8066	−1.8205	17.8285	5.04	4.78
12	perfluorobutane sulfonic acid	PFBS	300.10	0.8080	−2.8656	18.4512	1.75	1.77
13	perfluorohexane sulfonic acid	PFHxS	400.11	0.9019	−2.9066	18.4482	2.32	2.48
14	perfluoroheptane sulfonic acid	PFHpS	450.12	0.9099	−2.9179	18.4480	2.90	2.85
15	perfluorooctane sulfonic acid	PFOS	500.13	0.9159	−2.9214	18.4484	3.28	3.21
16	perfluorodecane sulfonic acid	PFDS	600.14	0.9253	−2.9291	18.4500	3.60	3.93
17	perfluorooctane sulfonamide	PFOSA	499.14	0.4709	−2.4774	18.2169	4.06	3.86
18	6:6 perfluoroalkyl phosphinic acid	6:6 PFPiA	702.07	0.6955	−2.2028	18.0435	4.93	4.94
19	8:2 perfluoroakyl unsaturated carboxylate	8:2 FTUCA	458.10	0.7245	−2.9775	17.8394	4.10	4.13
20	n-ethyl perfluorooctane sulfonamidoacetic	N-EtFOSAA	585.23	1.2244	−2.2628	18.1177	3.36	3.14
21	6:2 chlorinated polyfluorinated ether	6:2 Cl-PFAES	532.58	1.3756	−2.8813	18.5775	2.60	2.44
22	6:2 fluorotelomer phosphate diester	6:2 diPAP	790.17	0.7763	−0.8725	17.7807	4.51	4.55

References

1. Guelfo, J.L.; Higgins, C.P. Subsurface Transport Potential of Perfluoroalkyl Acids at Aqueous Film-Forming Foam (AFFF)-Impacted Sites. *Environ. Sci. Technol.* **2013**, *47*, 4164–4171.
2. Pi, N.; Ng, J.Z.; Kelly, B.C. Uptake and elimination kinetics of perfluoroalkyl substances in submerged and free-floating aquatic macrophytes: Results of mesocosm experiments with Echinodorus horemanii and Eichhornia crassipes. *Water Res*. **2017**, *117*, 167–174.
3. Chen, X.W.; Zhu, L.Y.; Pan, X.Y.; Fang, S.H.; Zhang, Y.F.; Yang, L.P. Isomeric specific partitioning behaviors of perfluoroalkyl substances in water dissolved phase, suspended particulate matters and sediments in Liao River Basin and Taihu Lake, China. *Water Res*. **2015**, *80*, 235–244.
4. Chen, M.; Wang, Q.; Shan, G.Q.; Zhu, L.Y.; Yang, L.P.; Liu, M.L. Occurrence, partitioning and bioaccumulation of emerging and legacy per- and polyfluoroalkyl substances in Taihu Lake, China. *Sci. Total Environ*. **2018**, *634*, 251–259.
5. Hu, H.M.; Zhang, Y.Y.; Zhao, N.; Xie, J.H.; Zhou, Y.Q.; Zhao, M.R.; Jin, H.B. Legacy and emerging poly- and perfluorochemicals in seawater and sediment from East China Sea. *Sci. Total Environ.* **2021**, *797*, 149052.
6. Labadie, P.; Chevreuil, M. Partitioning behaviour of perfluorinated alkyl contaminants between water, sediment and fish in the Orge River (nearby Paris, France). *Environ. Pollut.* **2011**, *159*, 1452–1453.
7. Li, F.S.; Sun, H.W.; Hao, Z.N.; He, N.; Zhao, L.J.; Zhang, T.; Sun, T.H. Perfluorinated compounds in Haihe River and Dagu Drainage Canal in Tianjin, China. *Chemosphere*. **2011**, *84*, 265–271.
8. Higgins, C.P.; Luthy, R.G. Sorption of perfluorinated surfactants on sediments. *Environ. Sci. Technol*. **2006**, *40*, 7251–7256.
